# Effects of Exercise-Based Cardiac Rehabilitation on Risk Factors, Fitness, and Quality of Life in Patients Undergoing Percutaneous Coronary Intervention: Emergency Department Versus Outpatient Routes

**DOI:** 10.3390/healthcare13233097

**Published:** 2025-11-27

**Authors:** Tong Yang, Yongchul Choi, Jiyoung Lee, Yonghwan Kim

**Affiliations:** Department of Physical Education, Gangneung-Wonju National University, Gangneung 25457, Republic of Korea; 20248068@gwnu.ac.kr (T.Y.); ycchoi@gwnu.ac.kr (Y.C.)

**Keywords:** coronary heart disease, cardiac rehabilitation, emergency room, outpatient, percutaneous coronary intervention

## Abstract

**Background/Objectives:** Hospital visits for the treatment of coronary heart disease primarily consist of routine outpatient (OP) or unscheduled emergency room (ER) visits. This study compared the effectiveness of cardiac rehabilitation (CR) based on the routes of hospital visits after percutaneous coronary intervention (PCI). **Methods**: Only men who completed three CR center visits (baseline, 6 months, 12 months) during the 12 months after PCI were analyzed. A total of 300 male patients were analyzed, with 206 patients in the OP group and 94 in the ER group, and their socioeconomic status, blood lipids, blood pressure, volume of oxygen peak (VO_2_ peak), physical activity, and quality of life (QoL) were compared. This study is a retrospective analysis. **Results**: The ER group was older and had lower economic income than the OP group (*p* < 0.05). In the OP and ER groups, total cholesterol (OP −10.4%, and ER −8.6%) and low-density lipoprotein cholesterol (OP −10.9% and ER −8.2%) improved in the third visit compared to the first visit (*p* < 0.05). Additionally, VO2 peak (OP 16.5%, and ER 14.3%), physical activity (OP 25.1%, and ER 4.4%), and body fat (OP −5.8%, and ER −4.5%) ultimately improved in both groups (*p* < 0.05). The interaction effect by time and group showed that the OP group significantly improved compared to the ER group in terms of high-density lipoprotein cholesterol, VO_2_ peak, exercise duration, grip strength, physical activity, body fat, and quality of life (*p* < 0.05). **Conclusions:** The 12-month CR program tended to improve outcomes in both groups, but the OP group showed greater improvements in high-density lipoprotein cholesterol, VO_2_ peak, and QoL compared to the ER group.

## 1. Introduction

Cardiovascular disease is a leading cause of mortality worldwide, with incidence rates per 100,000 ranging from 432 in Eastern Europe to 73 in high-income Asia-Pacific countries [1]. Although the cardiovascular disease mortality rate decreased by 34.9% globally from 1990 to 2022, it is still increasing in Korea. From 2001 to 2018, cardiovascular-disease-related deaths in Korea increased from 44.9 to 74.5 per 100,000 individuals [1,2,3].

Coronary heart disease (CHD) is among the most prevalent cardiovascular disorders. It is considered a critical emergency, as it can lead to sudden death [4]. Percutaneous coronary intervention (PCI), predominantly involving stent insertion, has become the preferred treatment approach [5].

As the prevalence of CHD is increasing due to lifestyle factors such as exercise habits and dietary choices, patients are being encouraged to undergo treatment and adhere to health maintenance practices collectively known as cardiac rehabilitation (CR) [6,7]. The results of a 1-year follow-up study showed that those who participated in CR had a higher smoking cessation rate (64 vs. 50%) and frequency of physical activity (3.9 vs. 3.4 days) than non-participants [8]. A meta-analysis of randomized controlled trials showed that the relative risk of angina pectoris and restenosis was lowered to 0.24 and 0.10, respectively [9].

Cardiac patients are divided into those who undergo planned treatment and those who present to the emergency room (ER) with sudden chest pain at any time [10]. Those who present to the emergency room have a different experience than those who undergo planned cardiac treatment on an outpatient basis. Their psychological state tends toward elevated levels of depression and anxiety, with diminished quality of life (QoL). Individuals with heart disease exhibit higher rates of emergency room (ER) visits compared to those with other conditions, often receiving cardiopulmonary resuscitation shortly before death [11]. Eken et al. reported that among ER patients, rates of panic attacks ranged from 16% to 43%, while rates of anxiety and depressive disorders ranged from 23% to 57% [12]. McGrad et al. found that psychological conditions, such as depression, influenced CR participation [11].

Therefore, while there may be differences in the effectiveness of CR participation between patients undergoing PCI after a scheduled outpatient (OP) visit and those undergoing PCI after an unexpected emergency room (ER) visit, this aspect has not yet been studied. Consequently, this retrospective study aimed to investigate the completion rate of a CR program over approximately one year and compare CR participation and its effectiveness among completing patients based on their treatment pathway (OP or ER). Through this study, we sought to identify changes in physical, psychological, and clinical risk factors based on the treatment pathway and contribute to the development of detailed, tailored considerations for individualized CR programs.

## 2. Materials and Methods

### 2.1. Participants

**Figure 1:**
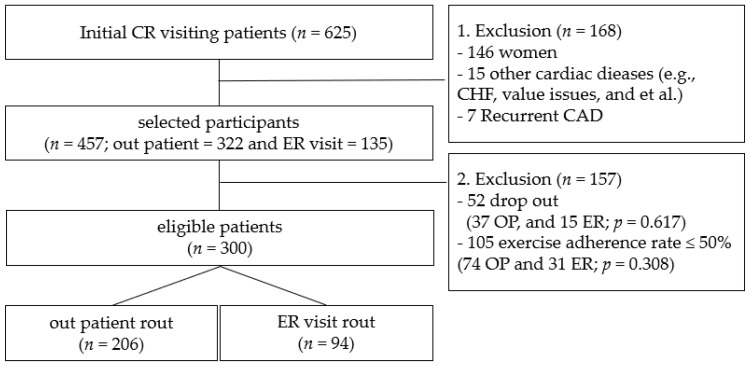
Inclusion and exclusion flow. *p* < 0.05; OP, outpatient; ER, emergency room; CR, cardiac rehabilitation; CHF, congestive heart failure; CAD, coronary artery disease.

Patients who underwent cardiac rehabilitation (CR) after PCI at a Korean hospital were monitored for 12 months. We started compiling data in January 2017. The first CR visit was to the cardiology department 3 weeks after PCI, and visits were made at 6 and 12 months. Primary data included lipid profiles, fitness assessments, and QoL questionnaires administered on the day of evaluation. Among those excluded were 141 women, 4 with congestive heart failure, 11 with valve disease, 7 with recurrent CHD, 157 with low exercise adherence, and 157 dropout patients (Figure 1). The participants provided voluntary consent for the analysis and possible future publication of their information. We included the STROBE Statement as Supplementary Materials.

### 2.2. Health Survey, Blood Collection, Quality of Life, and Physical Activity Questionnaire

The health survey collected the participants’ medical histories, including past and present illnesses, medications, surgeries, and treatments unrelated to heart disease. It also gathered information on social factors such as socioeconomic status (SES), occupation, and lifestyle habits like exercise, smoking, and drinking. The Korean version of the 36-item Short Form Health Survey (SF-36), a self-report questionnaire, was used to assess the participants’ health statuses [13]. The reliability of this questionnaire corresponds to a Cronbach’s alpha = 0.64, and the test–retest correlation coefficients range from 0.54 to 0.80 [14]. With a total of 36 questions, the participants chose the response option that best reflected their health status or experiences. Each item was scored out of 100 points, with higher scores indicating better health status.

The physical activity questionnaire used was the International Physical Activity Questionnaire developed by the World Health Organization (IPAQ) [15]. It is a self-recording method, and the amount of activity engaged in over the past week was recorded using a memory recall method. The Spearman Rho coefficients and Kappa values of test–retest reliability were 0.427–0.646 and 0.365–0.620 [16]. PA was assessed using an internationally accepted questionnaire [17], which relied on memory recall. However, the initial questionnaire captured PA patterns and habits prior to PCI treatment. This was necessary as patients were typically discharged with instructions limited to very-low-intensity brisk walking to facilitate heart stabilization post-treatment. The total PA level was calculated and recorded as metabolic equivalents (Mets)-min. IPAQ scores were calculated by multiplying duration (minutes/day), frequency (days/week), and MET values (walking 3.3 MET, moderate intensity 4.0 MET, vigorous intensity 8.0 MET). Total physical activity (MET-minutes/week) is the sum of these domains. Following guidelines, invalid values were excluded for extreme durations or unrealistic totals (>960 min/day), and incomplete or inconsistent responses were treated as missing values [18].

Blood was collected following an 8 h fasting period and at least 2 h prior to any treatment. A trained clinical pathologist performed blood collection, and analysis of commonly recognized cardiovascular risk factors, including total cholesterol, low-density lipoprotein cholesterol (LDLC), high-density lipoprotein cholesterol (HDLC), and triglycerides (TG), was conducted using an automated analysis facility.

### 2.3. Body Composition

Each patient underwent an InBody (InBody Inc., Seoul, Republic of Korea) measurement as the initial test at the CR center [19]. Preparation for testing involved taking measurements 2 h after eating, ensuring the bladder was emptied, and removing any metal accessories from the body. The patients were made to stand barefoot and use an alcohol swab to remove foreign substances before gripping the handle or electrode with their hands. Moisture was applied to prevent skin cells from being dry, which could hinder electrode permeability. During the test, which lasted approximately 1 min and 30 s, the participants’ arms and legs were slightly apart, and parameters such as body weight, body fat percentage, and muscle percentage were assessed.

### 2.4. Graded Exercise Tests (GXTs) and Grip Strength

For the test, we adhered to the Bruce protocol, with exercise intensity incremented every 3 min [20]. Volume of oxygen (VO_2_) levels were quantified using gas analysis (Vmax229, Sensormedics Co., Yorba Linda, CA, USA), and 12-lead electrocardiography (Case8000, GE Marquette Co., Milwaukee, WI, USA) was employed [21]. Before each intensity increase, chest pain etiology was assessed, and the rating of perceived exertion (RPE) was recorded. The cessation criteria comprised patient requests or the detection of electrocardiographic, blood pressure, or respiratory abnormalities. Maximal exercise capacity was indicated by a respiratory exchange rate exceeding 1.10 or an RPE score exceeding 15 [22]. VO_2_ values were measured using a mask equipped with a line and sensor in a breath-by-breath manner, with a 30 s average recorded in real time. After the test, the patients rested in a seated position until their HR dropped below 100 beats per min, with continuous monitoring. A 30 min interval was then allotted for result analysis and preparation of the CR program. Key parameters measured include VO_2_ peak and exercise time during the test.

Grip strength was measured using a grip dynamometer (Takei 5401; Takei Scientific Instruments, Niigata, Japan). Each patient was told to stand with their feet shoulder-width apart, straighten their back and chest, and look straight ahead. The patients were told to straighten their elbows and place their hands next to their thighs. The equipment was adjusted to fit each patient’s hand size, and each patient was instructed to grip as hard as possible while maintaining their posture after the start signal. Both hands were measured twice, and the average of both hands was recorded. Grip strength has long been used to measure muscle strength and exhibits a high correlation coefficient (r^2^ = 0.998) [23].

### 2.5. Exercise-Based CR Program

The CR program was developed based on the literature [24,25]. One program consisted of 15 min of stretching, 30–50 min of aerobic exercise, and 15 min of strength training. Stretching involved static stretches, with 15–20 repetitions held for 10 s; this process was repeated three times. Exercise intensity was set at 40–75% of VO_2_ reserve, in accordance with the American College of Cardiopulmonary Rehabilitation’s exercise prescription guidelines for heart disease patients. CR staff determined the optimal HR and exercise intensity based on the VO_2_ peak, overseeing training, practice, and monitoring. Intensity gradually increased from 40% in the first session to 60% in the third session or was set within the range of RPE 13–15. The aim for exercise volume was to exceed 500 Mets-min in total, and the exercises were conducted 3 to 5 times a week. Exercise adherence was recorded based on a subjective assessment of performance rate, with a target of three times per week.

### 2.6. Data Analysis

Data analysis was conducted using SPSS 25.0 (IBM Corp., Armonk, NY, USA). First, to test normality, the normal distribution was confirmed using the Shapiro–Wilk test for continuous variables. Descriptive statistics were utilized to calculate means and standard deviations for continuous variables, while frequencies and percentages were computed for categorical variables. An independent *t*-test was performed to compare the two groups at each visit, and a one-way ANOVA including a Bonferroni post hoc test was performed to compare the first, second, and third visit within the group. Additionally, a repeated two-way ANOVA was performed to assess temporal variations within each group. The predetermined threshold for statistical significance was set at *p* < 0.05.

## 3. Results

### 3.1. General Characteristics

The patients in the ER group were older compared to the OP group and had lower income. The OP group had a higher proportion of college graduates and employed individuals compared to the ER group (*p* < 0.05). However, there were no significant differences in alcohol consumption, smoking, number of blood vessels treated, or region of residence (*p* > 0.05) (Table 1). There were no differences between groups in the presence of pre-existing conditions such as hypertension, diabetes, and dyslipidemia, nor in the use of prescription medications (*p* > 0.05).

### 3.2. Comparison of Changes in Blood Pressure and Lipid Profiles

**Figure 2:**
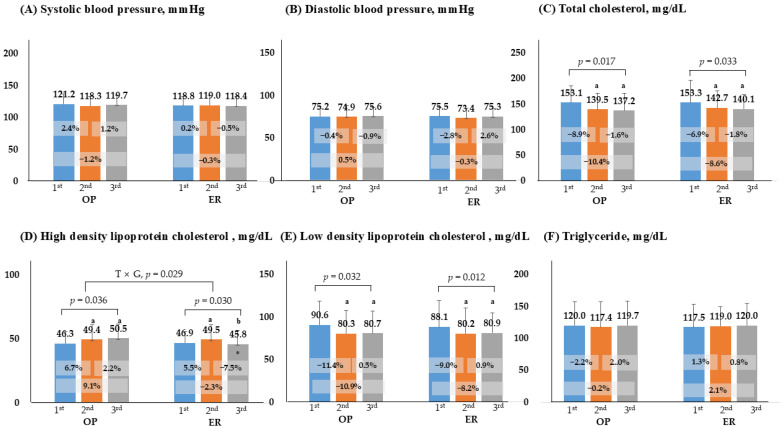
Comparison of changes in blood pressure and lipid profiles. *p* < 0.05; a, compares with 1st, b, compares with 2nd; *, significance between groups at each visit; OP, outpatient; ER, emergency room; T × G, time × group.

Blood lipids and blood pressure, for which increased levels are recognized as risk factors for cardiovascular disease, were not significantly different either between or within groups in terms of systolic blood pressure (SBP), diastolic blood pressure (DBP), and TG across the first, second, and third tests (*p* > 0.05). The OP and ER groups showed significant decreases in TC (−10.4% and −8.6%) and LDL-C (−10.9% and −8.2%) at the third visit compared to the first visit, while HDLC increased (13.4% and 5.5%). Meanwhile, the interaction effect by time and group was observed only in HDLC (*p* = 0.029) (Figure 2).

### 3.3. Comparison of Changes in Fitness, Physical Activity, and Body Composition

**Figure 3:**
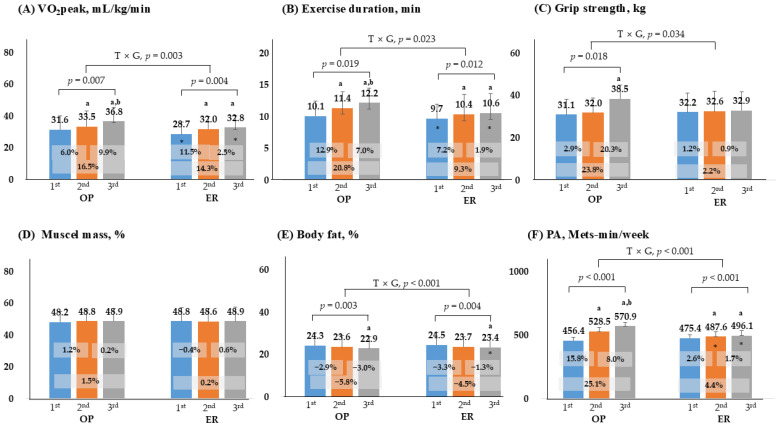
Comparison of changes in fitness, physical activity, and body composition. *p* < 0.05; a, compares with 1st, b, compares with 2nd; *, significance between groups at each visit; OP, outpatient; ER, emergency room; PA, physical activity; T × G, time × group.

Figure 3 presents the results regarding body composition, fitness tests, GXT, and PA. In the GXT, VO_2_ peak, and exercise duration were lower in the ER group than in the OP group in the first and third test. Grip strength remained unchanged in the ER group, and a significant difference was observed only in the OP group (first vs. third, 23.8%). Muscle mass showed no significant changes in either group (*p* > 0.05). Body fat decreased in both groups in the third test compared to the first (−5.8% and −4.5%), and there was a difference between groups in the third test (*p* < 0.001). According to the PA questionnaire, the ER group demonstrated increased activity levels in the second and third tests, although these levels were significantly lower than those in the OP group (*p* < 0.001). The interaction effect was significant for VO_2_ peak, exercise duration, grip strength, PA, and body fat (*p* < 0.05).

### 3.4. Comparison of Changes in Quality of Life

**Figure 4:**
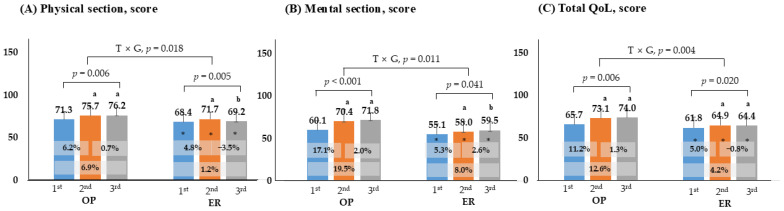
Comparison of changes in quality of life. *p* < 0.05; a, compares with 1st, b, compares with 2nd; *, Significance between groups at each visit; OP, outpatient; ER, emergency room; QoL, quality of life; T × G, time × group.

Figure 4 presents an evaluation conducted using QoL tools. The results showed that patients in the ER group consistently exhibited lower scores compared to those in the OP group across the first, second, and third tests (*p* < 0.05). For the physical and mental sections, both groups showed significant increases in the second assessment compared to the initial assessment. The interaction effect between time and group was significant, indicating that the OP group demonstrated a better QoL status than the ER group.

## 4. Discussion

In general, two treatment pathways exist for PCI. One route is for patients with OP to undergo planned PCI while receiving ongoing medical management for mild chest pain and related conditions such as dyslipidemia or hypertension. The second route is for individuals to experience unexpected chest pain and visit the emergency room. Particularly, patients visiting the ER and undergoing treatment often experience significant ongoing mental difficulties post-treatment. In this study, we focused on patients who underwent PCI via both pathways and compared the characteristics and effects of CR among those who completed a 12-month CR program.

In our study, individuals who dropped out of CR showed no significant difference in OP and ER, and this finding was similar to previous studies. In a study by Martin et al., both ER visitors and those treated according to a scheduled plan completed cardiac rehabilitation at similar rates, and dropout rates were also similar [26]. Resurreccion et al. conducted a comprehensive analysis of CR non-participation and dropout, examining various personal, social, and environmental factors [27], and it has been reported that even among those who initiate CR, only half successfully complete the intended program [11]. Thus, to enhance CR participation rates, healthcare professionals must understand the circumstances surrounding patients who have been treated in ER.

One of the major findings of this investigation was that the patients from the ER were older, had lower income, and were less educated than those in the OP group. Additionally, individuals in the ER reported lower QoL scores and demonstrated reduced levels of aerobic fitness. We believe that these findings could be inherently interconnected. Although OP patients frequently visit the hospital due to a need for chronic healthcare, they may also exhibit increased interest in their health and possess greater financial and temporal flexibility for treatment. Conversely, individuals who cannot afford healthcare may be vulnerable, even with respect to visiting the ER due to the development of fatal symptoms [28,29]. These findings align with those of Dawson et al., who found that individuals with a lower SES were more likely to visit the emergency room for chest pain and receive treatment [30]. Moreover, Herlitz et al.’s investigation revealed SES disparities in ER visits for chest pain, indicating that patients with lower income experience prolonged diagnostic delays compared to their higher-income counterparts. This difference could be due to financial considerations, particularly regarding the cost of diagnostic tests, with additional expenses likely required for expensive interventions such as PCI [31,32].

Individuals with a lower SES also exhibit lower rates of exercise, engagement in PA, and participation in leisure pursuits essential for consistent health management [33]. A study involving elderly individuals in China further underscored this relationship, revealing a mediating effect of 64.1% between education level and exercise participation [34]. Despite the availability of cost-free leisure activities like walking or running, individuals with lower incomes often encounter constraints in time and opportunities for enhancing fitness through PA compared to their more affluent counterparts [35]. Age constitutes a significant factor in these dynamics. As evidenced by our findings, the ER group tended to be older and had a higher prevalence of unemployment. Notably, the issue of elderly poverty is particularly acute in Korea, where retirement and unemployment constrain economic capacity, inevitably impacting access to healthcare [36]. Health managers need to focus more on individuals with low SESs in CR programs. Research by Sherrie et al. revealed that low-SES patients participate in CR at lower rate; these individuals are 5.8 times more likely to smoke compared to those with a high SES and show higher levels of depression, body mass index values, glycated hemoglobin levels, and waist circumferences. Conversely, they tend to have poorer cardiorespiratory health and physical performance [37].

One characteristic of the ER group identified in this study is the observation that, despite positive improvements noted in the second test, there was limited variation in several variables during the third round. Notably, while scores for HDLC and QoL exhibited an increase in the second round, they regressed to first-test levels in the third test. Similarly, metrics such as VO_2_ peak, exercise duration, and PA volume increased in the second test but plateaued in the third session, without further improvement. These trends, all pertaining to exercise-related variables, suggest a consistent pattern. Aksović et al. investigated the relationship between aerobic training and enhancements in fitness, finding that both moderate and high-intensity training contribute to improvements in VO_2_ levels. The most significant improvements were observed after six weeks of training, with sedentary individuals experiencing a higher rate of increase than active individuals [38]. These findings align with the results of our study. Following PCI, the initial visit’s low activity levels during the stabilization period likely contributed to low VO_2_ peak levels. Subsequent visits, particularly the second and third rounds, likely coincided with psychological recovery and increased engagement in PA, leading to either the maintenance or elevation of VO_2_ peak levels. The typical effects of participating in cardiac rehabilitation include management of risk factors and improvement in physical strength. In a study by Kerrigan et al., VO_2_ peak improved from 16.9 to 18.5 in patients with severe heart failure after 10 weeks of training, while the general management group showed a slight decrease [39].

This change difference in fitness may be related to the greater changes reported in the PA questionnaire among OP compared with ER. In this study, the PA questionnaire was administered during the initial visit, which carries a considerable risk of recall error. The questionnaire assesses activity levels over the last 7 days; however, recall-based surveys conducted after a time lag are susceptible to both overestimation and underestimation, a bias that may be particularly evident among patients. Watkinson et al. also reported that individuals with lower BMIs tended to overestimate their physical activity when completing PA questionnaires [40].

A notably positive outcome of this study was the observed positive changes in QoL mental scores within both groups; these changes were sustained over the long term, and they were particularly significant among patients with CHD. Rao et al. highlighted the prevalence of psychological distress among heart patients, with 18% experiencing moderate depression, 28% exhibiting anxiety, and 13% manifesting stress syndrome. Long-term follow-ups revealed a notable trend wherein individuals experiencing psychological discomfort had an increased likelihood of discontinuing CR, with the probability rising from 3.3 to 4.5 times [41]. Furthermore, individuals who had engaged in ER visits demonstrated a higher likelihood of panic disorder, anxiety, and depression compared to general patients [12]. Researchers have emphasized the importance of early exercise-based participation in cardiac rehabilitation (CR). In a study by Shan et al., patients who underwent coronary artery bypass grafting and participated in early cardiac rehabilitation demonstrated greater improvements in the 6 min walk, peak VO_2_, and anaerobic threshold compared to the control group. In addition, physical function, physical role, general health, and emotional role surveyed in the quality of life questionnaire also significantly improved [42].

In this study, non-significant findings concerning blood pressure and TG levels were observed, which could be attributed to the influence of prescribed medications, such as beta-blockers, dyslipidemia medications, and thrombolytics administered post-PCI treatment. Unfortunately, since medication adherence was not investigated in this study, it is not possible to strictly distinguish the effects of exercise participation from those of the medication. However, over the long term, TC and LDLC levels decreased in both groups, aligning with findings previously documented in several studies regarding the enduring effects of participation in CR [43,44].

Despite the valuable insights obtained from this study, several limitations must be acknowledged. A major limitation is the exclusion of a considerable number of patients from the analysis. In the case of Korean women, the incidence of cardiovascular disease tends to increase as much as in men as they age [2]. In light of this, research on women is very important, but to minimize heterogeneity in variables such as VO_2_ peak and grip strength, which differ by gender, female patients were excluded from the analysis. While this approach improves internal consistency, it limits the generalizability of the results to male PCI patients. Furthermore, excluding women limits the ability to identify gender-specific responses to cardiac rehabilitation. In future research, it would be very meaningful to design a study comparing women and men. This study included 12 months of data, but even this analysis could only examine completion and dropout rates among individuals who initially visited CR. Therefore, it was impossible to determine the number of those who never visited from the outset. This study has limitations as a retrospective study. Since it utilized previously collected clinical data, information may be missing or incomplete, which could affect the accuracy of the results. Furthermore, because participants were not prospectively controlled, there are limitations in establishing causality. Additionally, there is a possibility that researcher selection bias may have occurred. Some participants completed the CR program three times, but exercise compliance was very low. Furthermore, because it relied on subjective judgment and self-monitoring, there are limitations in explaining its accuracy. In addition, despite efforts to encourage participation through educational materials such as mobile or paper brochures, concerns remained regarding the feasibility of practice, especially for elderly individuals.

Clinically, the results of this study will provide information that is applicable in practice. The ER rout patients were elderly, low-income, and unemployed. Furthermore, they showed less improvement than the OP group fitness and exercise-related variables. These results will serve as reference information for clinical experts to establish health management strategies that enable ER patients to participate in CR and practice self-management. Moreover, quality of life in the ER group was consistently lower than that in the OP group. Therefore, it is the responsibility of health professionals to manage not only physical aspects but also mental well-being.

In future research, it would be useful to apply analyses that highlight the characteristics of female patients or examine the different trends between women and men through gender comparison. Future research should focus on evaluating recurrence and mortality rates, which are essential outcomes of CR, and monitoring long-term psychological and physical health maintenance. Additionally, it would be more fruitful to study CR participation rates, dropout rates, and mortality and recurrence rates in a comparative study of OP and ER patients.

## 5. Conclusions

Among male PCI patients who completed exercise-based CR, outcomes differed by hospital visit route. ER patients were older, had lower SES, and showed reduced aerobic fitness, PA levels, and QoL. Both groups demonstrated improvements in TC, HDLC, and LDLC at the third test compared with baseline. Significant interaction effects were observed for HDLC, VO_2_peak, exercise duration, PA amount, body fat, and QoL, with the OP group showing greater gains than the ER group across all assessments. These findings suggest that ER-route PCI patients require targeted management addressing lipid profiles, physical function, and overall QoL.

## Figures and Tables

**Table 1:** Clinical characteristics of the patients in the groups.

Variables	OP (n = 206)	ER (n = 94)	*p*
Age, years	58.0 ± 10.2	62.1 ± 9.9	0.022
Body mass index, kg/m^2^	24.8 ± 2.5	25.1 ± 2.4	0.396
Monthly income, USD	5495 ± 2329	4121 ± 2648	0.002
Education			
Middle school	40 (19.4%)	33 (35.1%)	<0.001
High school	105 (51.0%)	49 (52.1%)	
College or university	61 (29.6%)	12 (12.8%)	
Smoking			
Never smoker	46 (22.3%)	22 (23.4%)	0.735
Past smoker	87 (42.2%)	43 (45.7%)	
Current smoker	73 (35.4%)	29 (30.9%)	
Alcohol			
High risk	42 (20.4%)	24 (25.5%)	0.527
Medium risk	71 (34.5%)	33 (35.1%)	
Low risk	93 (44.7%)	37 (39.4%)	
Occupation			
Yes	128 (62.1%)	46 (48.9%)	0.032
No	78 (37.9%)	48 (51.1%)	
Residence region			
Area within the same city	129 (62.6%)	51 (54.3%)	0.170
Province	77 (37.4%)	43 (45.7%)	
Vessel number			
One	128 (62.1%)	54 (57.4%)	0.276
Two	60 (29.1%)	26 (27.7%)	
Three	18 (8.7%)	14 (14.9%)	
Pre-existing diseases			
Diabetes	73 (35.8%)	43 (44.8%)	0.162
Hypertension	136 (66.7%)	62 (64.6%)	0.794
Dyslipidemia	88 (43.1%)	51 (53.1%)	0.109
Prescribed medication			
Antiplatelet therapy	195 (95.6%)	89 (92.7%)	0.408
Statin	166 (81.4%)	79 (82.3%)	0.848
β-blocker	191 (93.6%)	86 (89.6%)	0.247
ACE inhibitors	183 (89.7%)	89 (92.7%)	0.525

*p* < 0.05; OP, outpatient; ER, emergency room; USD, United State Dollar; ACE, angiotensin-converting enzyme.

## Data Availability

The data presented in this study are available on reasonable request from the corresponding author due to privacy and ethical considerations.

## Supplementary Materials

The following supporting information can be downloaded at https://www.mdpi.com/article/10.3390/healthcare13233097/s1, Table S1: STROBE Statement—checklist of items that should be included in reports of observational studies.
